# Farmyard manure, a potential organic additive to reclaim copper and *Macrophomina phaseolina* stress responses in mash bean plants

**DOI:** 10.1038/s41598-023-41509-3

**Published:** 2023-09-01

**Authors:** Sundus Akhtar, Amna Shoaib, Iqra Javiad, Uzma Qaisar, Raazia Tasadduq

**Affiliations:** 1 Department of Plant Pathology, Faculty of Agricultural Sciences, University of the Punjab, Quaid-e-Azam Campus, Lahore, Pakistan; 2 School of Botany, Minhaj University Lahore, Lahore, Pakistan; 3 Central Park Medical College, Lahore, Pakistan; 4 School of Biological Sciences, University of the Punjab, Lahore, Pakistan; 5 Department of Biochemistry, Kinnaird College, Lahore, Pakistan

**Keywords:** Biochemistry, Microbiology, Plant sciences, Environmental sciences

## Abstract

In the era of global warming, stress combinations instead of individual stress are realistic threats faced by plants that can alter or trigger a wide range of plant responses. In the current study, the cumulative effect of charcoal rot disease caused by notorious fungal pathogen viz., *Macrophomina phaseolina* was investigated under toxic levels of copper (Cu) in mash bean, and farmyard manure (FYM) was employed to manage stress. Therefore, Cu-spiked soil (50 and 100 mg/kg) was inoculated with the pathogen, and amended with 2% FYM, to assess the effect of intricate interactions on mash bean plants through pot experiments. Results demonstrated that the individual stress of the pathogen or Cu was more severe for morpho-growth, physio-biochemical, and expression profiles of stress-related genes and total protein in mash bean plants as compared to stress combinations. Under single Cu stress, a significant amount of Cu accumulated in plant tissues, particularly in roots than in upper ground tissues, while, under stress combination less Cu accumulated in the plants. Nonetheless, 2% FYM in soil encountered the negative effect of stress responses provoked by the pathogen, Cu, or both by improving health markers (photosynthetic pigments, reducing sugar, total phenolics) and oxidative stress markers (catalase, peroxidase, and polyphenol oxidase), together with regulating the expression of stress-related genes (catalase, ascorbate peroxidase, and cytokinin-resistant genes), and proteins, besides decreasing Cu uptake in the plants. FYM worked better at lower concentrations (50 mg/kg) of Cu than at higher ones (100 mg/kg), hence could be used as a suitable option for better growth, yield, and crop performance under charcoal rot disease stress in Cu-contaminated soils.

## Introduction

Pakistan is the second largest importer of pulses in the world, while mash bean [*Vigna mungo* (L.) Hepper] is the third largest and most highly praised pulse crop in the country after chickpea and lentil^1^. In addition to being a major source of least expensive protein (22–24%) and energy, mash bean also provides substantial amounts of oil (2.1%), fats (1–2%), raffinose oligosaccharide (31–76%), carbohydrates (50–60%), and vitamins (A and B)^2^. Being a short-duration and drought-tolerant crop, it can be cultivated as emergency vegetation, especially in the rainfed area, which can make significant economic benefits to the farmers^1^. By and large, mash beans can be seeded after exhaustive crops e.g. wheat and rice to restore soil natural matter owing to their nitrogen-fixing ability, hence are considered fairly profitable^3^. Despite of good nutritive value and reasonable cost for consumers, the worldwide yield of mash beans including in Pakistan is very poor, which has decreased cultivation area in the country^1,2^. Among others, fungal diseases and heavy metals are undoubtedly the two most important stresses having a huge impact on the growth and productivity of crops. In this context, the charcoal disease caused by the soil-borne, necrotrophic fungus *Macrophomina phaseolina* (Tassi) Goid is the highest threat for pulses, especially in arid to tropical regions of the world, where it may cause 100% yield losses^4–6^. *M. phaseolina* is a notorious pathogen of over 500 economically valuable crops, causing various dry-weather wilts and rots, exhibits heterogeneous nature, is adaptable to diverse environmental conditions, and forms abundant microsclerotia which makes disease control challenging^7–9^. Up till now, there is no known vertical resistance (R-gene based) to *M. phaseolina*, and no systemic fungicides available to combat the menace^10,11^.

Routinely practiced and other human-dependent activities often containing heavy metals have increased the risk of fungal pathogen evolution and the jeopardy posed to them^12^. The soil surface is a fertile place for storing heavy metals, and then transferring them to the plants when they are highly concentrated, subsequently incorporated into the food chain and causing liver and brain disorders^13^. Among different metals, copper (Cu) mostly as CuSO_4_, is the most common naturally occurring compound accumulating in the soil through Cu-based fungicides, agricultural waste, agrochemicals, wood preservatives, tanning, sewage sludge, and other anthropogenic activities. High Cu in the soil (20–100 mg/kg) are known to impart toxic effects to soil microorganisms as well as plants and hinder the mineralization of macronutrients (N, P, and K) and micronutrients (Fe and Zn)^14^. In soil, 5–30 mg/kg and in plant tissue 3–10 mg/kg of Cu are regarded as normal^15^, where it contributes to hormone signaling, structural strengthening, photosynthesis process, and electron transport chain process in the plants^16^. However, higher Cu converts itself into complexes inside plant tissues, showing a high affinity for cell wall phenolic, carbonylic, carboxylic, sulfhydryl groups, nitrogen, oxygen, and sulfur atoms^17^. Excess Cu reduces plant growth and yield, decreases plant capacity to explore the soil for water and nutrient, alters root system design, while causing chlorosis, and necrosis, and induces lipid peroxidation and protein oxidation by acting as pro-oxidant^3,18^. So far, *M. phaseolina* can tolerate Cu toxicity due to higher osmotic pressure in the cell structure, while mycelial growth inhibition and morphological disorders were also recorded with increasing Cu concentration from 25 to 100 ppm, which can transform its viability and sporulation^19^. It is worth to mention at elevated concentrations, heavy metals not only alter the activities of harmful soil microorganisms but also affect beneficial flora which may also contribute to a reduction in soil fertility^20^. Thus, the concurrent occurrence of Cu with *M. phaseolina* either aggravates or inhibits the effect of the latter leading to either enhanced or reduced susceptibility to pathogens^21^. In this connection, 5–20 ppm of Cu has been reported to show the hormetic effect, while ≥ 40 ppm induces a toxic effect on plants under biotic stress^22^. Therefore, it is important to address the effect of both stress/s on plants simultaneously along with effective countermeasures to address both stresses^23,24^.

Organic soil amendments, including farmyard manure (FYM), are generally utilized as a cheaper and easily available fertilizer to supply macro- and micro-nutrients and restore the soil’s physical and chemical qualities in Pakistan^4^. FYM is a decomposed mixture of dung, urine, litter, and leftover materials from roughages and fodder fed to animals. A well-decomposed FYM contains 0.5–1.5% N, 0.2–0.4% P_2_O_5_, and 0.5–1.0% K_2_O, thus acting as a rich source of many nutrients (N, P, K, Ca, Mg, S, Zn, Fe, and Mn) in soil and plants^18^. FYM helps to manage soil-borne disease by improving the natural suppressiveness of the soil through generating microclimates unfavourable for pathogens, releasing fungitoxic compounds (e.g. ammonia nitrous acid), increasing competition against pathogens for resources, reducing the sclerotial number, and minimizing the inoculum contact with plant roots^25^. Furthermore, FYM acts as a sink, which reduces the mobility of metals in soil by adsorbing and volatilizing them, hence reducing their uptake by plants^26^. Apart from the role of FYM in disease and heavy metal stress tolerance in plants, FYM-mediated defense responses against stress combinations still need to be explored.

Recent shreds of evidence suggest that plants evolved multiple and interconnected signaling pathways to regulate different sets of stress-responsive genes resulting in diverse physiological and metabolic responses to confer tolerance to environmental stresses^24^. These include pathogenesis-related (PR) proteins, transcription factors, and enzymes involved in producing metabolites and hormones. Many efficient antioxidative systems work against reactive oxygen in coordination. Enzymes like superoxide dismutase (SOD), catalase (CAT), peroxidase (POX), and polyphenol oxidase (PPO) are known to act against stress^6,8^. These enzymes may be manipulated, overexpressed, or down-regulated depending on the strength of stress, duration, enzyme type, plant species, etc. Various workers have reported increased activities of many antioxidant enzymes in plants to overcome the oxidative stress induced by biotic or abiotic stresses^27^. In all cases, these enzymes are encoded by multiple gene families and are present in several compartments of the cells. Few of the genes including catalase (CAT), ascorbate peroxidase (APX), and cytokinin resistant 1 (CYR1) are expressed as a result of cellular responses in plants against stress^28^. CAT gene (heme peroxidases) translates the hydrogen peroxide to water and reduces the ROS (reactive oxygen species) levels to shelter the cells’ death by inducing a hypersensitive response under biotic stress^29^ and enhancing tolerance under metal-induced oxidative stress in the plants^30^. APX genes (heme peroxidase and copper oxidase family) are also involved in several stresses while acting against hydrogen peroxide in ascorbic acid and glutathione cycles, when it is overexpressed, the sugarcane plants show significant disease and Cu tolerance^31,32^. CYR1 (cryptochrome family) is involved in cytokinin signal transduction and activates R gene promoters in developing MYMIV-resistance in susceptible *Vigna* sp.^33^ and cytokinin modulate patterning—helps to escape from heavy metal stress^34^. Based on reports, it is crucial to assess alteration in activities of stress-related metabolizing antioxidant enzymes and their gene expression profiles to elucidate tolerance levels in mash bean under individual and combined stress of charcoal rot disease and Cu.

The current study aimed to investigate polygonal interaction of plant-pathogen-heavy metal along with the role of FYM on disease, physiological, growth, and molecular responses of of mash bean plants. The findings of the present study will be helpful in the management of charcoal rot disease of mash bean by natural, easily available, cheap sources, especially under the abiotic stress of Cu.

## Results

### Morphological and anatomical responses to single and combined pathogen and metal stress

**Figure 1:**
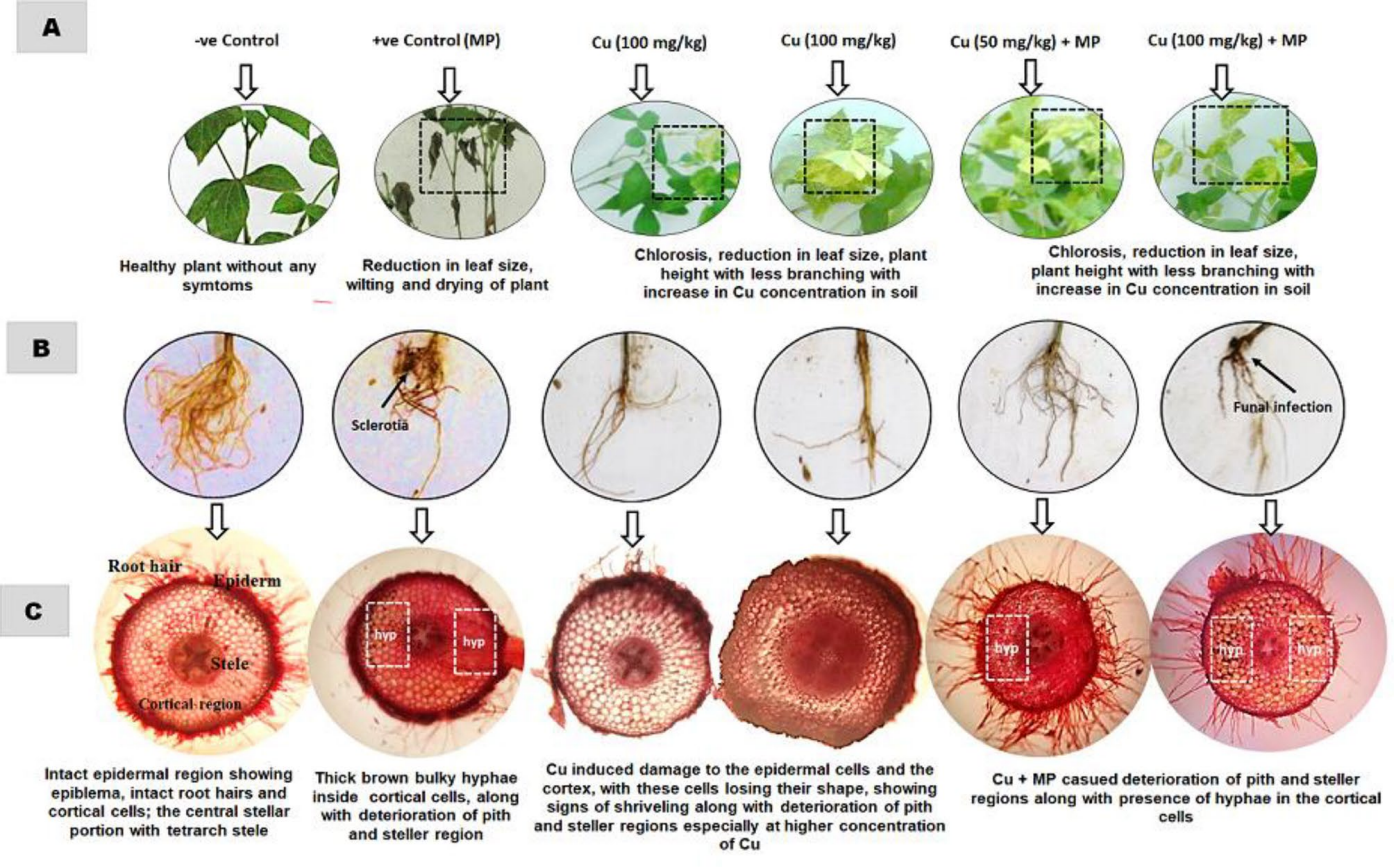
(**A–C**) Morphological and anatomical alterations in mash bean plant due to effect of *Macrophomina phaseolina* (MP) and Cu at 90th days of sowing. Symptoms of leaves (**A**); roots (**B**); and cross-section of the root (**C**).

Morphological observation showed that mash bean plants in the negative control treatments (T_1_) were healthy and asymptomatic. The infected plants in T_2_ (*M. phaseolina*) exhibited necrotic lesions and black sclerotial bodies on roots and stems accompanied by plant wilting and drying (Supplementary Data File). The morphological symptoms of Cu toxicity were evidenced by the chlorosis of the leaves in T_3_ (50 mg/kg) and T_4_ (100 mg/kg). Under combined stress in T_5_ (50 mg/kg + pathogen) and T_6_ (100 mg/kg + pathogen), there were fewer symptoms of disease and Cu toxicity. In all treatments (T_2_-T_6_), there was a marked reduction in growth (leaf area, height, and root area) as well (Fig. 1A,B).

Cross-section of mash bean root showed that the individual and combined stress of *M. phaseolina* or Cu rendered abnormalities in root cells in comparison to T_1_, where all the epidermal cells were intact, the cortex was homogeneous, stele included the central core of vascular tissue in a tetrarch condition, and the root hairs were turgid. Infected roots in T_2_ clearly presented thick brown bulky hyphae inside cortical cells, pith, xylem, and phloem accompanied by disintegration of pith and steller regions. Exposure to Cu in T_3_ and T_4_ caused abnormal cell shape, loss of tissue differentiation, and damage to epidermal cells, cortex, pith, and steller regions, while the changes were more evident at the higher Cu concentration (100 mg/kg). The combined stress pattern was similar to the pathogen stress, where fungal hyphae were presence in cortical cells and there was deterioration of pith and steller regions (Fig. 1C).

### Effect of FYM on survival, morpho-growth, and yield responses in mash bean subjected to single and combined pathogen and metal stress

There was 100% survival of healthy plants in the negative control (T_1_), while the pathogen-inoculated plants (T_2_) exhibited a significantly (p ≤ 0.05) greater disease severity index (100%) with higher plant mortality (86%). Application of 2% FYM significantly managed 75% of diseases and enhanced plant survival to 81% in T_8_. The stress of single Cu (T_3_) or in combination with the pathogen (T_5_) was managed completely by the application of 2% FYM in T_9_ and T_11_. However, single or combined stress with higher Cu concentration (T_4_ and T_6_) was mitigated up to 80% in T_10_ and T_12_ (Table 1).

**Table 1:** Effect of soil amendment with Farmyard manure (FYM) on disease and metal toxicity in mash bean due to *Macrophomina phaseolina* (MP) under Cu toxicity.

Treatments	Disease severity index (%)	Plant mortality (%)	Yellow patches on leaves due to Cu toxicity (%)
T_1_: − ve control	0.0^f^	0.0^e^	0.0^f^
T_2_: + ve control (*Macrophomina phaseolina*, MP)	100^a^	87.0^a^	–
T_3_: Cu (50 mg/kg)	–	0.0^e^	33.0^c^
T_4_: Cu (100 mg/kg)	–	0.0^e^	60.0^a^
T_5_: Cu (50 mg/kg) + MP	61.0^b^	41.0^b^	12.0^c^
T_6_: Cu (100 mg/kg) + MP	49.0^c^	38.0^b^	38.0^b^
T_7_: 2% FYM	–	0.0^e^	–
T_8_: 2% FYM + MP	25.0^d^	19.0^c^	–
T_9_: 2% FYM + Cu (50 mg/kg)	–	0.0^e^	3.0^f^
T_10_: 2% FYM + Cu (100 mg/kg)	–	0.0^e^	17.0^e^
T_11_: 2% FYM + Cu (50 mg/kg) + MP	2.0^f^	0.0^e^	0.0^f^
T_12_: 2% FYM + Cu (100 mg/kg) + MP	15.0^e^	15.0^d^	13.0^c^

Different letters (superscript) in the column depict significant differences (p ≤ 0.05) as determined by LSD Test.

**Figure 2:**
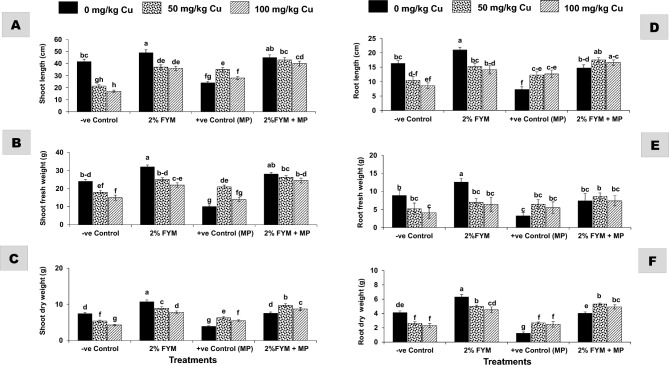
(**A–F**) Effect of 2% FYM on vegetative growth-related attributes of mash bean under separate and simultaneous stress of *Macrophomina phaseolina* (MP) and excess copper (Cu) at 90th days of sowing. Vertical bars show standard errors of means of six replicates. Values with different letters at their top show significant difference (p ≤ 0.05) as determined by LSD-test.

**Figure 3:**
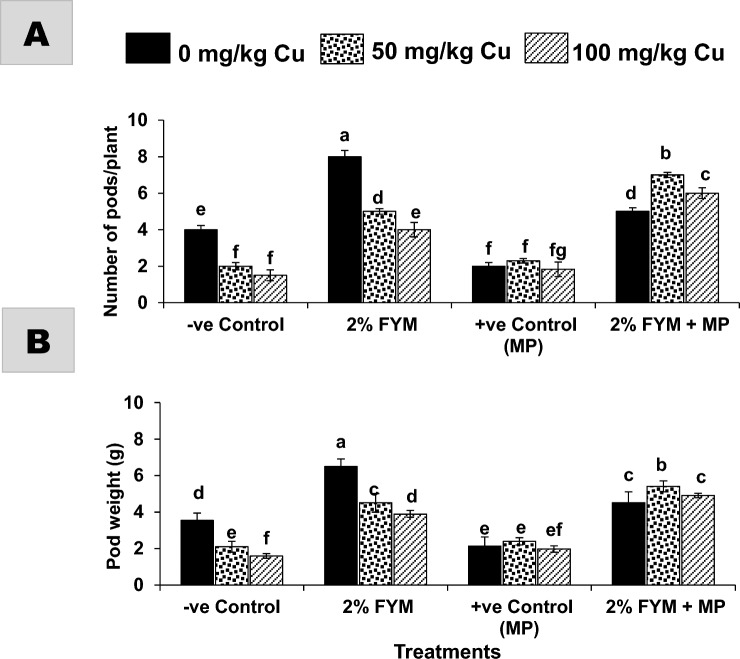
(**A**) and (**B**) Effect of 2% FYM on yield-related attributes of mash bean under separate and simultaneous stress of *Macrophomina phaseolina* (MP) and excess copper (Cu) at 90th days of sowing. Vertical bars show standard errors of means of six replicates. Values with different letters at their top show a significant difference (p ≤ 0.05) as determined by LSD-test.

One-way ANOVA revealed that each stress, alone or in combination, significantly affected plant growth and yield, though, single stress resulted in even more detrimental effects, however, the application of 2% FYM managed the stress to a great extent (Fig. 2A–F). In particular, the single stress of pathogen or metal decreased all growth and yield attributes (length, fresh and dry weight of shoot and root) to a similar extent by ~ 30–60% as compared to T_1_. The stress combination in T_5_ significantly decreased only root and yield indices by 30% over T_1_, whereas all growth measurements were twofold greater (p ≤ 0.05) than T_2_, and insignificantly differing with respect to the corresponding metal treatment in T_3_. The combined stress at higher Cu (100 mg/kg) in T_6_, had 20–40% fewer measurements of the growth and yield indices than T_1_, but 30–100% more than T_2_ and the corresponding metal treatment in T_4_. Soil amended with 2% FYM significantly enhanced all attributes of growth either in the absence/presence of any stress by 50–150% in different treatments as compared to the corresponding control treatments. Moreover, the improvements in many treatments reached statistically the same range comparable to the T_1_ (Figs. 2A–F, 3A,B).

**Figure 4:**
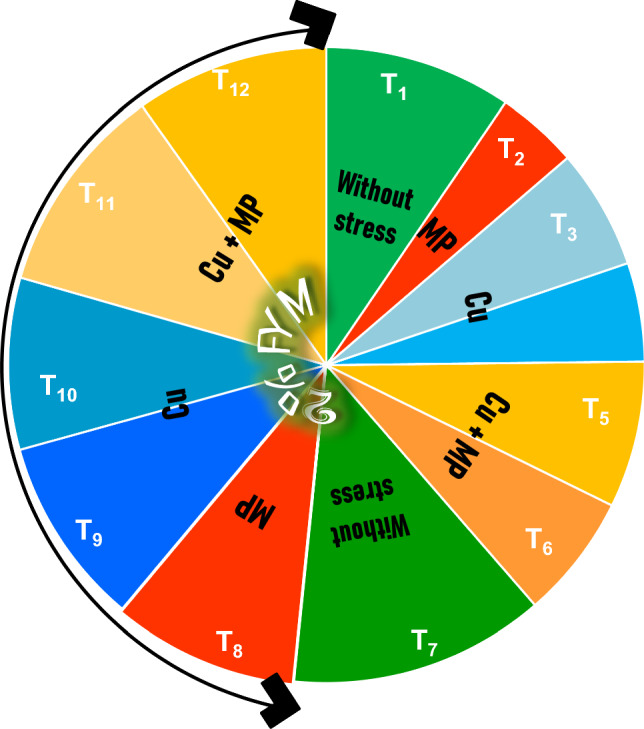
Cumulative growth tolerance indices in mash bean including vegetative growth attributes of mash bean plants exposed to separate and simultaneous stress of *Macrophomina phaseolina* (MP) and excess copper (Cu) at 90th days of sowing.

T_I_ (tolerance index) was calculated by taking the growth and yield attributes as an indicator to determine the capability of a plant to grow in a stressful environment. Results revealed that the T_1_ of mash bean plants was 1 in negative control, and reached to minimum value of 0.44 when flourishing under the biotic stress of *M. phaseolina* followed by 0.53 and 0.64 under abiotic stress of 100 mg/kg and 50 mg/kg Cu, respectively. The T_I_ of mash bean plants under stress combinations in T_5_ (0.78) and T_6_ (0.67) was less than the negative control, but greater as compared to pathogen or corresponding metal treatments. However, soil manuring with 2% FYM elevated T_I_ in all treatments with the highest value of 1.37 in T_7_, followed by 1.13, 1.03, 1.01, 0.99, and 0.92 in T_8_, T_9_, T_10_, T_11_, T_8_, and T_12_, respectively (Fig. 4).

### Effect of FYM on physiological and biochemical responses in mash bean subjected to single and combined pathogen and metal stress

**Figure 5:**
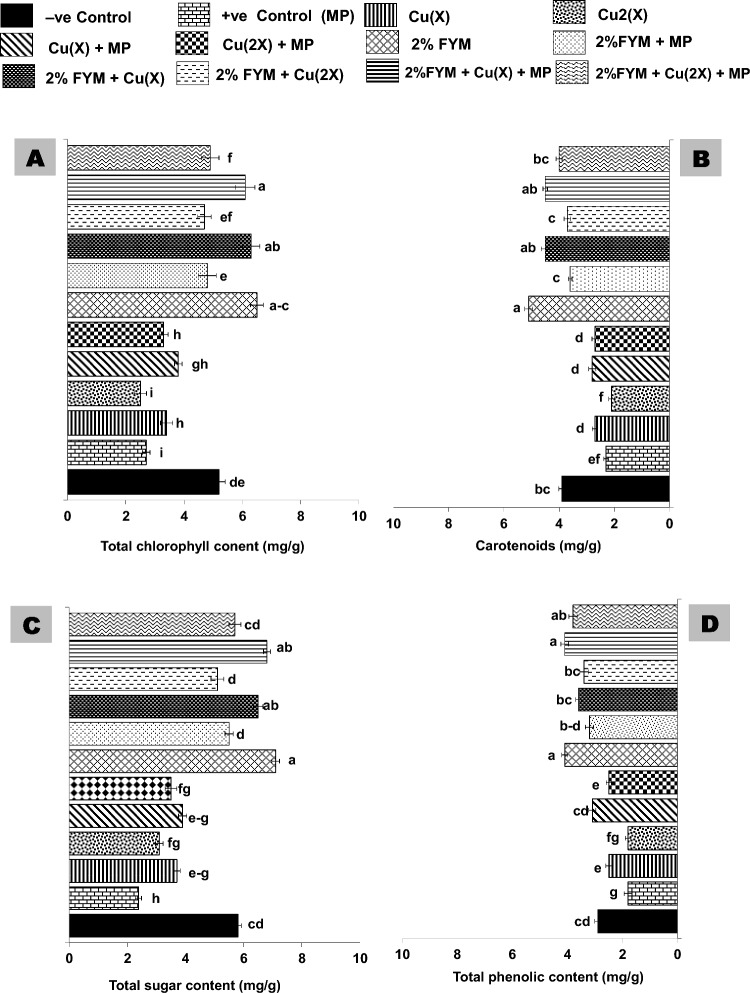
(**A-D**) Effect of 2% FYM on physiological attributes of mash bean leaf under separate and simultaneous stress of *Macrophomina phaseolina* (MP) and excess copper (Cu) at 45th days of sowing. Vertical bars show standard errors of means of six replicates. Values with different letters at their top show a significant difference (p ≤ 0.05) as determined by LSD-test.

There was a significant reduction of 30–50% in the total chlorophyll content (TCC), carotenoids (CR), reducing sugar (RS), and total phenolics (TPC) in all treatments (T_2_-T_6_) in comparison to negative control. Soil amendment with 2% FYM significantly (p ≤ 0.05) and variably improved the said attributes by 30–40%, 80–130%, 50–70%, 60–90%, 30–70%, and 50–60% in T_7_, T_8_, T_9_, T_10_, T_11_, and T_12_, respectively as compared to their corresponding control treatments. Furthermore, the effect of 2% FYM on physiological responses in T_7_-T_12_ was statically equal to or greater than T_1_ (Fig. 5A–D).

**Figure 6:**
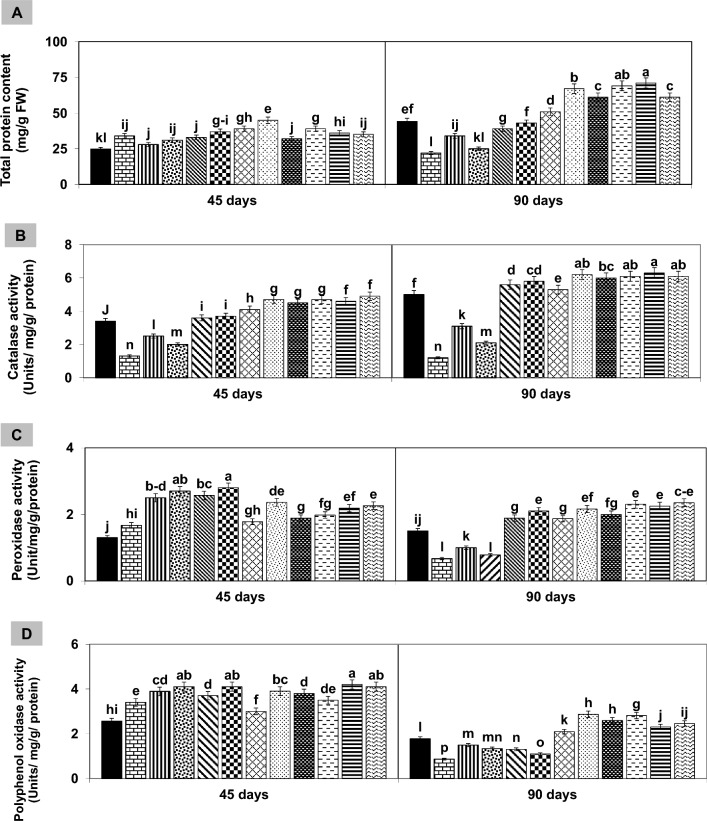
(**A–D**) Effect of 2% FYM on biochemical attributes of mash bean leaf under separate and simultaneous stress of *Macrophomina phaseolina* (MP) and excess copper (Cu) at 45th days of sowing. Vertical bars show standard errors of means of six replicates. Values with different letters at their top show a significant difference (p ≤ 0.05) as determined by LSD-test.

Biochemical responses in mash bean plants were analyzed twice (45 and 90 days after sowing, DAS). Results indicated that total protein content (TPC) and activity of antioxidant enzymes (POX and PPO) improved initially at 45 DAS, then declined at 90 DAS. The activity of CAT activity decreased under single stress treatments, while improved under stress combination at either growth stage. The soil application with 2% FYM significantly enhanced these attributes variably up to 1.5–twofold in all treatments at both growth stages (Fig. 6).

### Effect of FYM on expression levels of genes in mash bean subjected to single and combined pathogen and metal stress

**Figure 7:**
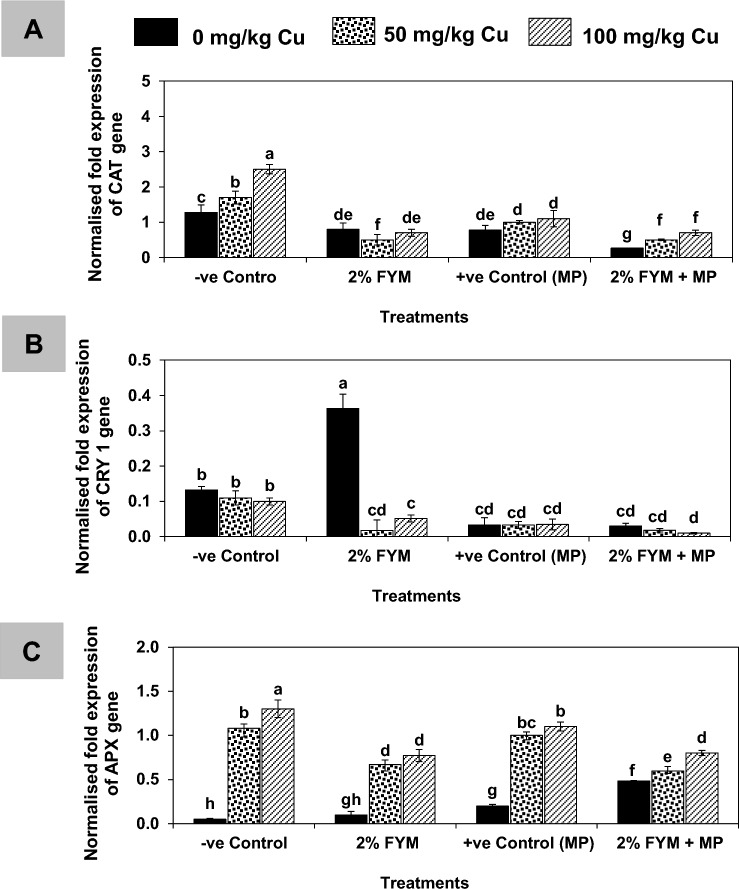
(**A–C**) Quantitative analysis of catalase (CAT), cytokinin-resistant genes (CYR1) and ascorbate peroxidase (APX) expression levels in mash bean leaf upon treatment with 2% FYM, *Macrophomina phaseolina* (MP) and excess copper (Cu) at 45th days of sowing. Vertical bars show standard errors of means of six replicates. Values with different letters at their top show a significant difference (p ≤ 0.05) as determined by LSD-test.

The findings of real-time PCR indicated, that the expression levels of CAT and CYR1 genes were significantly decreased in the positive control of mash bean plants by 40% and 70%, respectively, while that of ascorbate peroxidase substantially improved by many folds as compared to the negative control. In treatments provided with 50 and 100 mg/kg Cu, the expression level of CYR1 changed insignificantly, however, the expression levels of CAT improved significantly by 22% and 89%, respectively and that of ascorbate peroxidase improved by 95% and 250%, respectively relative to the negative control. The stress combination insignificantly changed the expression level of the CAT gene, decreased the CRYI gene, and enhanced ascorbate peroxidase significantly. The maximum expression level of the CYR1 gene was recorded in negative control after soil amendment with 2% FYM. In comparison, it was significantly decreased in the rest of the treatments supplemented with 2% FYM. Likewise, 2% FYM also reduced the expression levels of CAT and ascorbate peroxidase genes in all treatments as compared to the negative control (Fig. 7).

### Effect of FYM on protein profile in mash bean subjected to single and combined pathogen and metal stress

**Figure 8:**
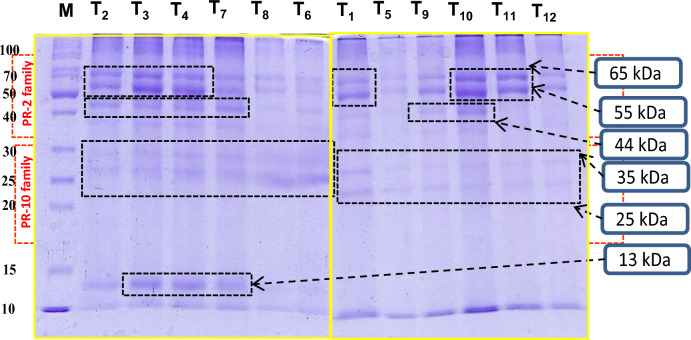
Sodium dodecyl sulphate–polyacrylamide gel electrophoresis (SDS-PAGE) for 45-days old mash bean leaf due to the effect of soil amendment with 2% FYM on charcoal rot disease caused by *Macrophomina phaseolina* (MP) and excess copper (Cu) at 45th days of sowing. T_1_: − ve Control; T_2_: + ve Control (MP); T_3_: Cu (50 mg/kg); T_4_: Cu (100 mg/kg); T_5_: Cu (50 mg/kg) + MP; T_6_: Cu (100 mg/kg) + MP; T_7_: 2% FYM; T_8_: 2% FYM + MP; T_9_: 2% FYM + Cu (50 mg/kg); T_10_: 2% FYM + Cu (100 mg/kg); T_11_: 2% FYM + Cu (50 mg/kg) + MP and T_12_: 2% FYM + Cu (100 mg/kg) + MP. Yellow boxes indicate the grouping of gels cropped from different parts of the same gel.

Relative to the negative control, considerable changes in the electrophoretic profiles (**~ **10 kDa to 65 kDa) of mash bean leaves were observed in other treatments (Supplementary Data File). For instant, protein bands at ~ 55 kDa and 65 kDa were identified in all treatments, though these were intensified in response to individual stress of pathogen or metal. The expression levels of the band at 55 kDa were further increased by 4–5 times due to the application of 2% FYM under Cu stress. There was up-regulation of expression of the protein band at ~ 44 kDa in all treatments without 2% FYM, and the expression was further intensified 3–4 times after the soil amendment with 2% FYM. Bands at ~ 25–35 kDa were up-regulated in single and stress combination treatments while down-regulated after adding 2% FYM. New bands at ~ 13 kDa were also observed under single and stress combination treatments, while absent in other treatments (Fig. 8).

### Effect of FYM on Cu accumulation in mash bean subjected to single and combined pathogen and metal stress

**Figure 9:**
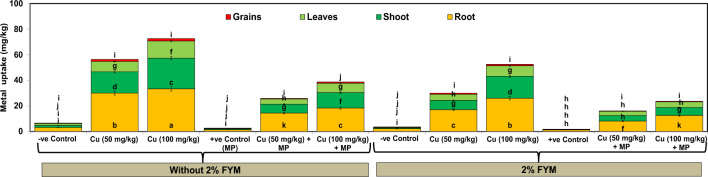
Copper (Cu) uptake by different parts of mash bean due to the effect of soil amendment with 2% FYM, *Macrophomina phaseolina* (MP), and excess Cu at 90th days of sowing. Vertical bars show standard errors of means of six replicates. Values with different letters at their top show a significant difference (p ≤ 0.05) as determined by LSD test.

Soil fortification with Cu resulted in an increase in Cu accumulation by different plant parts in order of root > stem > leaves > grains in a concentration-dependent manner (Fig. 9). Therefore, when Cu (50 and 100 mg/kg) alone was added to soil, roots accumulated 53–65% of Cu followed by stem (30–33%), leaves (16–18%) and grains (1–2%). In stress combination, the plant parts accumulated less Cu, and the accumulation rate ranged between 28–30%, 16–17%, 8–10%, and 0.8–1% by root, stem, leaves, and grain, respectively. After mixing of 2% FYM in soil, the plant accumulated half time less Cu (Fig. 9). Translocation factor (TF) and bioaccumulation factor (BAF) values (0.88–1.18 and 3.56–5.32, respectively) revealed an increase in accumulation and subsequent translocation of Cu to above-ground parts from the root under Cu stress, while TF and BAF values (0.79–1.11 and 1.48–2.36, respectively) indicating limited accumulation and later translocation to shoot and grains from the underground part in Cu + pathogen treatments. Over and above, soil application with 2% FYM caused more translocation of the metal in the soil as indicated by low values of TF and BAF as compared to their respective control treatments (Table 2).

**Table 2:** Translocation factor and bioconcentration factor of mash bean leaf due to the effect of soil amendment with 2% FYM under *Macrophomina phaseolina* and Cu stress.

Treatments	TF	BF
T_1_: − ve control	0.70	0.15
T_2_: + ve control (*Macrophomina phaseolina*, MP)	0.70	0.18
T_3_: Cu (50 mg/kg)	0.88	3.56
T_4_: Cu (100 mg/kg)	1.18	5.32
T_5_: Cu (50 mg/kg) + MP	0.79	1.48
T_6_: Cu (100 mg/kg) + MP	1.11	2.36
T_7_: 2% FYM	0.20	0.01
T_8_: 2% FYM + MP	0.43	0.07
T_9_: 2% FYM + Cu (50 mg/kg)	0.75	1.10
T_10_: 2% FYM + Cu (100 mg/kg)	1.02	2.13
T_11_: 2% FYM + Cu (50 mg/kg) + MP	0.98	0.54
T_12_: 2% FYM + Cu (100 mg/kg) + MP	0.87	0.88

### Multivariate analysis

**Figure 10:**
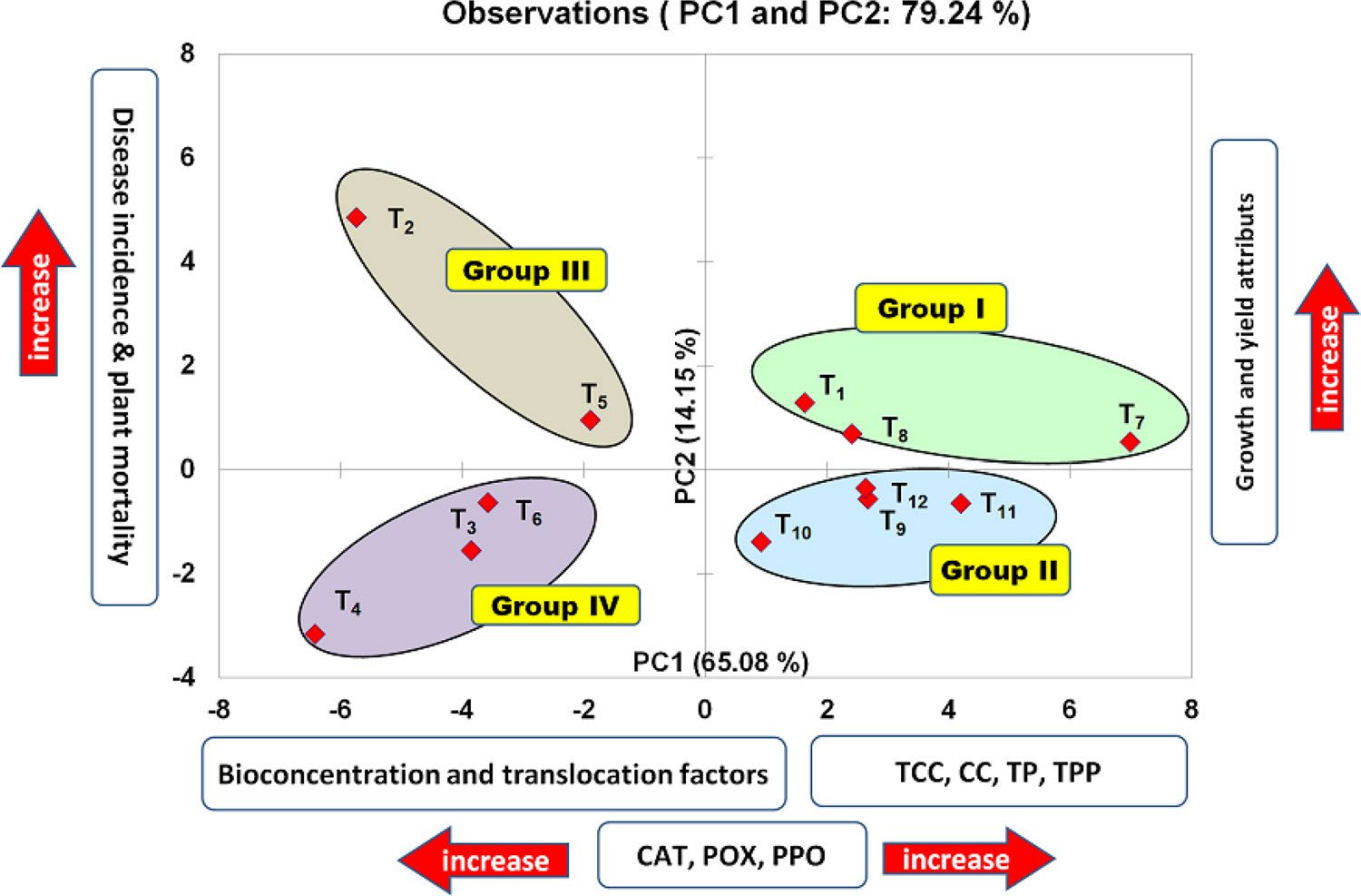
Principal component analysis of biophysical, biochemical, molecular and metal accumulation attributes in mash bean plants due to the effect of soil amendment with 2% FYM, *Macrophomina phaseolina* (MP), and excess copper (Cu). *TCC* total chlorophyll content, *CC* carotenoids, *TRS* total reducing sugar, *TP* total phenolic, *TPP* total protein content, *CAT* catalase, *POX* peroxidase, *PPO* polyphenol oxidase. T_1_: − ve Control; T_2_: + ve Control (MP); T_3_: Cu (50 mg/kg); T_4_: Cu (100 mg/kg); T_5_: Cu (50 mg/kg) + MP; T_6_: Cu (100 mg/kg) + MP; T_7_: 2% FYM; T_8_: 2% FYM + MP; T_9_: 2% FYM + Cu (50 mg/kg); T_10_: 2% FYM + Cu (100 mg/kg); T_11_: 2% FYM + Cu (50 mg/kg) + MP and T_12_: 2% FYM + Cu (100 mg/kg) + MP.

A PCA was performed to identify the association of variables with each other and their effect on the treatments. The PCA-biplot aided in finding the best-performing treatments in response to each stress alone or in combination providing 2% FYM to manage stress. The data summarized in Fig. 10 showed that PC1 and PC2 explained 79.24% of the variability in the data (Fig. 10). In the PCA, the treatments residing more on the left side exhibited sensitivity of the treatments to the given stress (pathogen, metal, and their combinations) with inferior values of the morpho-physiological traits. The treatments positioned at the right edge were found to be appropriate treatments for stress alleviation and for better morpho-physiological traits. Therefore, on the basis of acquired data, the biplot was divided into four distinct groups. Group I in the upper right side of biplot consisted of control (T_1_) along with treatments that received 2% FYM (T_7_ and T_8_) followed by treatments (T_9_, T_10_, T_11_, and T_12_) in the lower right side in group II. Highly sensitive treatments in the upper left (Group III: T_2_ and T_5_), and moderately sensitive treatments on the lower left side (Group III: T_3_, T_4_, and T_6_) of the biplot presented the level of sensitivity increased as treatments were placed away from the origin (Fig. 10).

## Discussion

The contemporary study was conducted to ascertain the impact of charcoal rot on mash beans under abiotic stress of excess Cu providing FYM as soil amendments against stress/s. Generally, morpho-growth, as well as, physio-molecular traits of mash bean plants were more prone to individual stress of *M. phaseolina* and excess Cu, than the stress combinations.

Infection caused by *M. phaseolina* has declined the growth, and yield of mash bean plants significantly by 30–70%, inciting 100% disease incidence and 86% plant mortality. The infected plants exhibited necrotic lesions, wilting, and drying of the whole plant. Anatomical features of mash bean root also revealed the occurrence of fungal hyphae in epidermal cells passing through intercellular spaces of the cortex, causing the disintegration of vascular bundles possibly through the production of toxic compounds like phaseolinone by embedded sclerotia^11^. The current findings are in harmony with many previous reports, where *M. phaseolina* has deteriorated plant health by inciting charcoal rot disease in them^3,4,8^ due to the successful establishment of the pathogen within host tissue accompanied by the production of different toxins and cell wall degrading enzymes for disruption of the vascular system^11^. The host susceptibility to *M. phaseolina* may also be ascribed to suppression of the auxin response and alteration in the jasmonic acid and ethylene pathways by the pathogen^4^. *M. phaseolina*-induced reduction in the chlorophyll concentration was likely to be associated with a decrease in the stomatal conductance and photosynthesis, while the increase in respiration rate and other metabolic pathways involved in defense mechanisms together with the enhanced movement of metabolites to the fungal cells^4^. Likewise, the reduction in physiological markers and alerted activity of CAT, POX, and PPO could be associated with low levels of resistance in the host plants due to disturbance in electron flow at the membrane-bound organelle^11^.

Cu-excess (50 and 100 mg/kg) condition strongly impaired mash bean morpho-physiological responses by reducing these attributes (30–60%), induced foliar chlorosis with reduced leaf area, shoot branching, size, and the number of pods, however, no mortality was recorded in the plants. Damage to root meristem, thickening, and cracking of the root cuticle were also observed^35^. Similar findings have been reported earlier due to Cu phytotoxicity, where excess Cu imbalanced uptake of essential elements (N, P, K, Ca, Mg, Fe, and Zn), affected the cell membrane of the root cuticle, altered the auxin homeostasis, cell division, cell expansion, cell elasticity, and elongation, while decreased growth and biomass production in the plants^36,37^. The stress combination led to fewer symptoms of disease or Cu toxicity, accompanied by less reduction in the growth and yield indices which could be the result of additive, synergistic and antagonistic effects of the single stress^38^. The changes in growth and yield indices were consequences of altered host physiology as revealed by the reduction in the physiological markers and enchantment in the biochemical markers. Exposure to Cu in soil fosters the production of ROS, which possibly by reducing the efficiency of the photosynthetic process interfere with the chlorophyll organization and functionality, causing the reduction of electron transport and PSII activities hence decreasing chlorophyll a, b, carotenoids, photosynthetic gas exchange, photosystem II and PSII quantum yield^36^. Below a certain value of Cu, the synthesis of low molecular weight stress proteins reinforced the action of the antioxidant enzymes^36,38^,. Adrees et al.^39^ reported that the increasing CuSO_4_ caused a dose-dependent increase in ROS generation due to enhancing electrolyte leakage which boosted the activities of superoxide dismutase (SOD) and other antioxidants (CAT and POX). Liu et al.^31^ in another study indicated that a toxic level of Cu for 24 h triggers the MAPK (mitogen-activated protein kinases) pathway in *Zea mays* causing an enhancement in the activities of SOD, CAT, and APX. In another study, Younis et al.^40^ exhibited enhancement in SOD and CAT activities under low doses of Cu, but their activities declined under high levels of Cu in *Phaseolus vulgaris*. Liu et al.^32^ also documented an increase in SOD and POX activities at 5 mg/L concentration of Cu, while declining in the CAT activity in *Salvinia natans*.

The tolerance indices of the mash bean plants in terms of growth and yield parameters were 0.4, 0.5–0.6, and 0.7–0.8 with *M. phaseolina*, Cu, and Cu + *M. phaseolina*, respectively, which revealed that the plants experienced stress with values < 1. However, under combined stress mash bean plants probably developed tolerance due to the hormesis effect^41^, which could lead to adaptive responses of organisms to moderate environmental challenges, improving their functionality and/or tolerating stronger challenges in the future resulting in improvement in the activity of enzymes^20^. Hence, it is plausible to suppose that, high Cu not only affects plants but also pathogens under stress combinations involving Cu and *M. phaseolina*, which was further evident by the reduction in the translocation factors and bioaccumulation factors under combined stress. Moreover, greater accumulation of Cu in the root followed by the stem, leaves, and grains could be ascribed to efficient metal efflux through the plasma membrane, chelation of Cu with organic molecules, stimulation of phytochelatins, metallothioneins and heat shock proteins in roots may restrict upward movement from roots to aerial parts^42^.

Soil mixing with 2% FYM substantially improved growth and yield in mung bean plants under stress and unstressed conditions, which resulted in high tolerance indices (> 1) in the different treatments^4^. The nutritional profile of sandy loam soil (sand: 42%, silt: 32%, and clay: 25%) indicated it contained a sufficient amount of organic matter (6.14%) and other nutrients (N: 0.29%, P: 0.03%, and K: 0.21%, respectively), which were deficient in bare soil (organic matter: 0.4%; N: 0.01%, P: 0.001% and K: 0.01%, respectively). The nutrients in 2% FYM possibly furnished abounding organic matter for the growth and development of beneficial microorganisms, hence improving root architecture, supporting dissolution, and nutrient availability to mash bean plants^18^. Therefore, the 2% FYM possibly by increasing the resources for the self-protection of plants, may lead to restoration in chloroplast-to-nucleus communication, therefore accounting for improvement in crop yield. Therefore, the mash bean plants, exposed to single or simultaneous stress in the presence of 2% FYM exhibited an increase in physiological and biochemical markers presenting metabolic cost for limiting the adverse effects of *M. phaseolina*, Cu, or their combinations^4^. Over and above, the plant accumulated half-time less Cu in the presence of 2% FYM due to the strong sorption of Cu to the soil organic matter.

The enhancement in the genes of expression of CAT and APX under separate and simultaneous stress of *M. phaseolina* and Cu may indicate the antagonistic action of CAT and APX against the overproduction of H_2_O_2_^43^. The reduction in the expression of CAT and APX after soil amendment with 2% FYM could be ascribed them as highly regulated genes for induction of compensatory mechanisms in mash beans against stress environment^44^. CYR1 gene responded positively to 2% FYM under stress conditions by showing the highest expression levels probably due to the higher uptake of nutrients from the soil with the greater increase in root length. Moreover, CYRI gene has been noticed to regulate genes involved in root growth indicating an important function of genes in regulating root growth^45^.

An increase in total protein content may specify mash bean plant could withstand a stressed environment^24^. Protein profiling acquired through SDS-PAGE reflected several changes in the 45-day mash bean leaf relative to the negative control. Many bands at approximately 13 kDa, 25 kDa, 35 kDa, 44 kDa, 65 kDa, 55 kDa, and 44 kDa were observed with greater intensity in the treatments containing *M. phaseolina* or Cu. Kieffer et al.^46^ documented abundance of PR proteins class I chitinases (27–28 KDa; PR-3 family), several β-1,3-glucanases (PR-2 family), and thaumatin-like protein (PR-5 family) in cadmium exposed poplar leaves. Likewise, an increase in the expression of PR proteins (10–40 kDa) against heavy metal stress was correlated with adaptation to stressful environments^47^. Furthermore, greater intensity of protein bands at 37–50 kDa (PR-2 family) in plants might be responsible for inhibiting fungal growth through the disintegration of cell wall chitin and glucagon^24^. By contrast, the expression level of the protein at 37–50 kDa decreased under stress combination could be the result of cross-tolerance mechanisms, and normalized in 2% FYM + Cu + *M. phaseolina* possibly owing to the enhancement of a plant's resistance against stresses.

PCA explained 79% of the data variability^8^. Factor-loading matrix extracted from biplot analysis of all PCA derived from growth, yield, physio-biochemical, molecular (gene expression), responses as well as through tolerance indices, translocation factors, and bioaccumulation factors indicated, a positive correlation of the growth and yield attributes of mash bean plants with 2% FYM soil amendments. Besides, the placement of treatments in group I and II near the control grouphighlighting the significance of 2% FYM as a soil amendment in alleviating single stress induced by *M. phaseolina* and Cu, and stress combinations. Therefore, soil amendment with 2% FYM could be utilized to alleviate the charcoal rot disease in mash bean plants growing under the toxic concentration of Cu (50 and 100 mg/kg).

## Materials and methods

### Experiment

The experiment was conducted during the period of May–July (average temperature: 40 ± 5 °C and average relative humidity 50 ± 5%) in pots kept in a tunnel in the Experimental Station of the Faculty of Agricultural Sciences, University of the Punjab Lahore, Pakistan. For the greenhouse assay, the soil was then sterilized by fumigation^8^. The Cu solutions (50 and 100 mg/kg) for spiking were prepared from CuSO_4_. 5H_2_O. The soil was spiked by spraying an aqueous solution of Cu with the continuous turning of the soil and left for 15 days for drying^24^. Decomposed 2% FYM was mixed in the measured amount of sterilized, sieved metal spiked soil, filled in pots (7ʺ × 6ʺ h × w, 5 kg/pot), and left for another 4 days. Fungal suspension for inoculation was prepared by growing the *M. phaseolina* (FCBP-0751) on 2% MEA medium kept at 28 °C until profuse sporulation occurred, usually in 4 to 7 days. The culture comprised of both pycnidia and sclerotia was harvested by scraping them with glass beads and suspended in 30 mL of distilled water. The sclerotial number in the suspensions was adjusted) by hemocytometer (Marienfeld GmbH, Marienfeld, Germany). The freshly prepared cultural suspension (100 mL, 2.0 × 10^5^ sclerotia/mL) was inoculated in each pot's upper 2–6 inch layer. The inoculated soil in each pot was left for 3 days under natural environmental conditions to establish the pathogen. Certified surface-sterilized seeds of mash bean var. Maikhaldia 6066 (provided by Ayub Agriculture Research Institute, Pakistan) was surface-sterilized with 1% Clorox for 5–10 min prior to washing with sterilized distilled water 3 times^4^. After drying, seeds were sown (10 seeds per pot), and 7 seedlings were maintained after a successful stand. The pots were placed in a completely randomized design with six replications (Table 3). All plants were kept inside a transparent plastic chamber to facilitate the infection process, and the experiment was intended for 90 days.

**Table 3:** Treatments designed for the current experiment.

Experiment treatments	
T_1_	− ve control
T_2_	+ ve control (*Macrophomina phaseolina*, MP)
T_3_	Cu (50 mg/kg)
T_4_	Cu (100 mg/kg)
T_5_	Cu (50 mg/kg) + MP
T_6_	Cu (100 mg/kg) + MP
T_7_	2% FYM
T_8_	2% FYM + MP
T_9_	2% FYM + Cu (50 mg/kg)
T_10_	2% FYM + Cu (100 mg/kg)
T_11_	2% FYM + Cu (50 mg/kg) + MP
T_12_	2% FYM + Cu (100 mg/kg) + MP

### Disease and Cu toxicity measurements

After 90 days of sowing, the mash bean plants were analyzed for Cu toxicity symptoms (percentage chlorosis or yellowing in foliage), disease severity index, plant mortality, and tolerance index (T_index_). The charcoal rot symptoms caused by the *M. phaseolina* on mash bean plants was assessed using 1–9 rating scale^48^.

$$\mathrm{Disease\, index }=\left[\mathrm{R\, }\left(\mathrm{rating\, }\times \mathrm{\, number \,of\, plants\, rated}\right)/\mathrm{Total\, number\, of\, plants\, }\times \mathrm{ highest\, rating}\right] \times 100$$

$$\mathrm{Mortality\, }(\mathrm{\%})=\frac{\mathrm{No}.\mathrm{ \,of\, plants \,died}}{\mathrm{Total\, no}.\mathrm{ \,of \,plant \,assessed}}\times 100,$$

$${\mathrm{G}}_{\mathrm{i}}=\left(\frac{{\mathrm{G}}_{\mathrm{x}}}{{\mathrm{G}}_{\mathrm{max}}}\right){\mathrm{T}}_{\mathrm{index}}= \sum \left(\frac{{\mathrm{G}}_{\mathrm{x}}}{{\mathrm{G}}_{\mathrm{max}}}\right){\mathrm{n}}^{-1},$$

where, G_i _= normalized growth parameter; G_x_ = individual growth or yield parameter; G_max_ = maximum value; T_index_ = Tolerance index.

### Anatomical assays

To examine anatomical changes in roots, the sections of the treated as well as control samples the roots were cut and prepared by several washing with 0.3, 0.5, 0.9, and 1% of alcohol and stained with 0.25% safranin (w/v, dissolved in 50% ethanol) for tissue differentiation. These sections were mounted in 20% glycerin to prepare temporary mounts and observed under a compound microscope and photographed with a digital imaging system^49^.

### Physio-biochemical assays

Physiological attributes like total chlorophyll content (TCC), carotenoids (CR), reducing sugar (RS), and total phenolics (TPC) were assessed in the leaf samples of 45 days old plants, while the total protein content (TPP) and activities of enzymes were assays in the leaf of 45 and 90-days-old mash bean plants.

### Total chlorophyll content (TCC), carotenoids (CR), reducing sugar (RS), and total phenolic content (TP)

The concentration of TCC (Chl a, Chl b, and CR), was assayed using the Lichtenthaler method^50^ against a blank of acetone at 646, 663, and 470 nm for Chl a, Chl b and CR, respectively. RS was measured in the leaf sample homogenized in ethanol (80%), centrifuged at 800 rpm for 10 min followed by the addition of arsenomolybdate reagent and measurement of the samples at 620 nm^51^ and using Folin–Ciocalteu method^52^, the TPC of the samples was measured at 760 nm against a blank using gallic acid as a standard.

### Total protein content (TPP) and antioxidant enzyme activities

Homogenized leaf samples in chilled sodium phosphate buffer (100 mM, pH 6.8) were analyzed for TPP and enzyme activities. Leaf extract containing reagents [(reagent A: sodium carbonate (Na_2_CO_3_) + NaOH (0.1 N) + sodium potassium tartrate) (reagent B: copper sulfate) (reagent C: mixing 50 mL reagent A with 1 mL of reagent B)] was mixed with the Folin-phenol reagent, incubated for 30 min, and quantified for TPC at 620 nm using as standard the bovine serum albumin^53^. The assay mixture for CAT activity (EC: 1.11.1.6) was measured at 240 nm, which contained buffer B (0.05 M sodium phosphate buffer (pH 7.0) and 0.036% hydrogen peroxide) and the enzyme extract. For POX activity (EC 1.11.1.7), the reaction mixture contained phosphate buffer (pH 7.0), enzyme extract, 5.33% pyrogallol, and 0.5% H_2_O_2._ The samples were measured for absorbance at 420 nm after incubation for 5 min at 25 °C^54^. PPO activity (EC 1.14.18.1), was determined at 495 nm in the reaction mixture made with 0.1 M sodium phosphate buffer (pH 7.0), 10 mM catechol, and enzyme extracr^55^.

### Gene expression by RT-PCR

For the gene expression study, the ground leaf powder was homogenized in extraction buffer, followed by shaking in a shaker incubator at 42 °C for 1.5 h. Later on, the samples were mixed with potassium chloride, centrifuged and the collected supernatant was mixed with lithium chloride. After overnight incubation, samples were centrifuged again to precipitate RNA pellets. Which was then washed with 2 M lithium chloride, mixed in 1/10 volume of 2 M potassium acetate, and incubated on ice to precipitate other unwanted contaminants. DNA-free total RNA (4 µg) was used to synthesize cDNA. For that, a kit of SuperScript^®^ III Reverse Transcriptase (Life Technologies, Inc) was used. For qRT-PCR, cDNA was further diluted, and 200 ng of cDNA was mixed with Maxima Sybr green qPCR master mix (Thermo Scientific, Inc.) + 250 nM of forward and reverse primers for each gene (Table 4). Amplification and detection of the product assays were carried out in iQ5 cycler (Bio-Rad, Inc.). Melting curve analysis was also performed to check the specificity of primers^56^.

**Table 4:** Sequences of primers used in qRT-PCR.

No	Primer name	Sequence (5’–3’)
1	ActinTF	ATTGAGCATGGATTGTGAG
2	ActinTR	GGCGACATACATAGCAGGAG
3	CatalaseTF	GCAAAGGGTTTCTTTGAGGT
4	CatlaseTR	GAAGACGGGAAGGTTGTTTC
5	CYR1 TF	AAAAGGTGCTTTGCTTATTGTG
6	CYR1 TR	TGCCAAGTCATTCAAAAGGT
7	Ascorbate peroxidaseTF	TTCGGAACCATCAAGCACC
8	Ascorbate peroxidaseTR	CTCAACTGCGACAACTCCAG

### Protein profiling by SDS-PAGE

Protein samples were run on 10% SDS-PAGE gels [(separating gel: 1.5 M Tris pH 8.8, 10% SDS (w/v), 30% (v/v) acrylamide, 10% (w/v) (NH_4_)_2_S_2_O_8_, 0.05% (v/v) TEMED; stacking gel: 1 M Tris pH 6.8, 10% (w/v) SDS, 30% acrylamide, 10% (NH_4_)_2_S_2_O_8_, 0.01% (v/v) TEMED)]. About 2 μL of protein was mixed with 8 μL 1X running buffer loading dye (60 mM Tris pH 6.8, 25% (v/v) glycerol, 5% (w/v) SDS, 1% (v/v) saturated bromophenol blue. After incubating for 30 min at room temperature, proteins were run in 1X SDS running buffer (250 mM Tris pH 8.3, 500 mM glycine, 1% [w/v) SDS] at 200 V with a protein size marker, until the dye was 1–2 mm from the end of the gel. Gel was stained in a Coomassie Blue stain solution (0.1% (w/v) Coomassie brilliant blue, 45% (v/v) methanol, and 10% (v/v) acetic acid) for 20–30 min and then washed with PAGE-destain [10% (v/v) acetic acid, 45% (v/v) ethanol] for several times to visualize protein bands.

### Growth, yield, and Cu analysis

After 90 days of sowing, lengths of shoot and root were measured in cm, while weights were computed in grams. Yield assays like the number of pods/plant and the weight of pods were also taken at the time of harvest. The dry mass of shoots and roots was recorded after keeping them in a scientific oven at 60 °C for 48 h.

The soil was analyzed for texture, organic matter, and macronutrients (nitrogen, potassium, and phosphorus) before and after mixing with 2% FYM according to methods explained by International Soil Reference and Information Centre^57^. For determination of total Cu concentration in the treatments provided with Cu, the dried soil, root, stem, leaf, and seeds samples of the plants were powdered, and digested separately using 2 mL 70% v/v nitric acid at 100 ºC for 2 h exposures and analyzed through Atomic absorption spectroscopy (Thermo scientific ICE 3000 SERIES). The translocation factor was calculated by the following equation (Cshoot and Croot are metal concentrations in the shoot and root of the plant, respectively. TF > 1 represents that the translocation of metals effectively was made to the shoot from the root)^58^. The bioconcentration factor was also calculated (SMC: shoot metal concentration, SDW: shoot dry weight; RMC: root metal concentration, RDW: roots dry weight)^59^.

$${\text{TF}}\,{ = }\,{\text{Cshoot/Cro}}\,{\text{ot}}$$

$$\mathrm{BCF }\left(\mathrm{\%}\right)=\mathrm{ SMC}\times \mathrm{SDW}+\mathrm{RMC }\times \mathrm{RDW }\left(\mathrm{A}\right)=\mathrm{ SMC}*\frac{\mathrm{RMC}}{\mathrm{A}}.$$

### Statistical analyses

Data were subjected to the LSD test (p ≤ 0.05), and Pearson correlation was used to analyze the correlation between metal accumulation in the plants and morpho yield-related attributes. Moreover, all the statistical analyses were done by using the computer software Statistics 8.1. Principal components analysis was performed to summarize the variability of the treatments and to determine the association among the measured traits.

### Ethical approval

All procedures in this experiment were carried out in accordance with relevant guidelines of the university field of the University of the Punjab, Lahore, Pakistan.

## Conclusions

The present experiment presented strong evidence that the single and combined stresses caused variations in the morpho-growth, yield, physio-biochemical, gene expression, and protein profiling in mash bean plants resulting in low tolerance indices. However, mash bean plants were more sensitive to the single stress of *M. phaseolina* or Cu than their combined stress. Under Cu stress, a significant amount of Cu accumulated in plant tissues, particularly in roots than in upper parts. However, under stress combination less Cu accumulated in the plants. Soil amendment with 2% FYM mitigated the individual and simultaneous stress factors by inducing resistance in mash bean plants through improving photosynthetic pigments, reducing sugar, total phenolics, and activity of antioxidant enzymes (CAT, POX, and PPO), regulating the expression of stress-related genes (CAT, APX, and CYR1), and proteins which induced greater biomass and productivity. Additionally, the application of 2% proved effective in reducing Cu-accumulation in the plants, as evidenced by a reduction in the translocation of Cu from roots to aerial parts as well as a decrease in TF and BAF. This data suggests that the application of 2% FYM to crops might be a valid strategy to overcome charcoal rot disease in mash bean in Cu-contaminated soils.

### Supplementary Information

Supplementary Figures.

## Data Availability

The datasets used and/or analysed during the current study available from the corresponding author on reasonable request.

## References

[1] Qayyum A, Iqbal J, Barbanti L, Sher A, Shabbir G, Rabbani G, Rafiq MK, Tareen MN, Tareen MJ, Amin BAZ (2019). Mash Bean [*Vigna*
*mungo* (L.) Hepper] Germplasm evaluation at different ecological conditions of Pakistan. Appl. Ecol. Environ. Res..

[2] Zia-Ul-Haq M, Ahmad S, Bukhari SA, Amarowicz R, Ercisli S, Jaafar HZ (2014). Compositional studies and biological activities of some mash bean (*Vigna*
*mungo* (L.) Hepper) cultivars commonly consumed in Pakistan. Biol. Res..

[3] Akhtar S, Shoaib A, Akhtar N, Mehmood R (2016). Separate and combined effects *of Macrophomina phaseolina* and copper on growth, physiology and antioxidative enzymes in *Vigna mungo* L. J. Anim. Plant Sci..

[4] Khan KA, Shoaib A, Arshad Awan Z, Basit A, Hussain M (2018). *Macrophomina phaseolina* alters the biochemical pathway in *Vigna radiata* chastened by Zn^2+^ and FYM to improve plant growth. J. Plant Interact..

[5] Shoaib A, Akhtar M, Javaid A, Ali H, Nisar Z, Javed S (2021). Antifungal potential of zinc against leaf spot disease in chili pepper caused by *Alternaria alternata*. Physiol. Mol. Biol. Plants.

[6] Shoaib A, Abbas S, Nisar Z, Javaid A, Javed S (2022). Zinc highly potentiates the plant defense responses against *Macrophomina phaseolina* in mungbean. Acta Physiol. Plant.

[7] Shoaib A, Nisar Z, Javaid A, Khurshid S, Javed S (2019). Necrotrophic fungus Macrophomina phaseolina tolerates chromium stress through regulating antioxidant enzymes and genes expression (MSN1 and MT). Environ. Sci. Pollut. Res..

[8] Shoaib A, Khan KA, Awan ZA, Jan BL, Kaushik P (2022). Integrated management of charcoal rot disease in susceptible genotypes of mungbean with soil application of micronutrient zinc and green manure (prickly sesban). Front. Microbiol..

[9] Khambhati VH, Abbas HK, Sulyok M, Tomaso-Peterson M, Shier WT (2020). First report of the production of mycotoxins and other secondary metabolites by *Macrophomina*
*phaseolina* (Tassi) Goid. isolates from soybeans (*Glycine*
*max* L.) symptomatic with charcoal rot disease. J. Fungi.

[10] Siddique S, Shoaib A, Khan SN, Mohy-Ud-Din A (2021). Screening and histopathological characterization of sunflower germplasm for resistance to *Macrophomina phaseolina*. Mycologia.

[11] Marquez, N., Giachero, M.L., Declerck, S., & Ducasse, D.A. *Macrophomina phaseolina*: General characteristics of pathogenicity and methods of control. *Front. Plant Sci.* **12***,* (2021) 10.3389/fpls.2021.634397. 10.3389/fpls.2021.634397 PMC8100579 33968098

[12] Pandaranayaka EP, Frenkel O, Elad Y, Prusky DB, Harel A (2019). Network analysis exposes core functions in major lifestyles of fungal and oomycete plant pathogens. BMC Genom..

[13] Taylor AA, Tsuji JS, Garry MR, McArdle ME, Goodfellow WL, Adams WJ, Menzie CA (2020). Critical review of exposure and effects: Implications for setting regulatory health criteria for ingested copper. Environ. Manag..

[14] Mir AR, Pichtel J, Hayat S (2021). Copper: Uptake, toxicity and tolerance in plants and management of Cu-contaminated soil. Biometals.

[15] Alengebawy A, Abdelkhalek ST, Qureshi SR, Wang MQ (2021). Heavy metals and pesticides toxicity in agricultural soil and plants: Ecological risks and human health implications. Toxics.

[16] Afzal A, Shafqat A, Akhtar S, Sultana T, Kazmi A, Ali A, Mashwani ZUR, El Askary A, Gharib AF, Ismail KA, Khalifa AS (2022). Biosorbents removed copper heavy metal from agricultural land cultivated with *Vigna radiata* (Mung bean). Int. J. Agron..

[17] Quartacci MF, Cosi E, Meneguzzo S, Sgherri C (2003). Uptake and translocation of copper in Brassicaceae. J. Plant Nut..

[18] Kumar V, Pandita S, Sidhu GP, Sharma A, Khanna K, Kaur P, Bali AS, Setia R (2021). Copper bioavailability, uptake, toxicity and tolerance in plants: A comprehensive review. Chemosphere.

[19] Akhtar S, Shoaib A (2020). The counter defence system of antioxidants in Coelomycetous emerging human and plant pathogenic fungus Macrophomina phaseolina against copper toxicity. Environ. Sci. Pollut. Res..

[20] Gajewska J, Floryszak-Wieczorek J, Sobieszczuk-Nowicka E, Mattoo A, Arasimowicz-Jelonek M (2022). Fungal and oomycete pathogens and heavy metals: An inglorious couple in the environment. IMA Fungus.

[21] Ramegowda V, Senthil-Kumar M (2015). The interactive effects of simultaneous biotic and abiotic stresses on plants: Mechanistic understanding from drought and pathogen combination. J. Plant Physiol..

[22] Morkunas I, Woźniak A, Mai VC, Rucińska-Sobkowiak R, Jeandet P (2018). The role of heavy metals in plant response to biotic stress. Molecules.

[23] Khurshid S, Shoaib A, Javaid A (2016). Chromium toxicity to tomato (*Lycopersicum esculentum* Mill.) susceptible to Fusarium wilt pathogen. Curr. Sci..

[24] Khurshid S, Shoaib A, Javaid A, Akhtar F, Shafiq M, Qaisar U (2017). Management of Fusarium wilt of tomato by soil amendment with *Cenchrus pennisetiformis* under chromium stress. Physiol. Mol. Plant Pathol..

[25] Mitsuboshi M, Kioka Y, Noguchi K, Asakawa S (2018). Evaluation of suppressiveness of soils exhibiting soil-borne disease suppression after long-term application of organic amendments by the co-cultivation method of pathogenic *Fusarium oxysporum* and indigenous soil microorganisms. Microbes Environ..

[26] Rani V, Bhatia A, Kaushik R (2021). Inoculation of plant growth promoting-methane utilizing bacteria in different N-fertilizer regime influences methane emission and crop growth of flooded paddy. Sci. Total Environ..

[27] Tariq M (2021). Role of nanoparticles in abiotic stress. AgriTech.

[28] Sofo A, Scopa A, Nuzzaci M, Vitti A (2015). Ascorbate peroxidase and catalase activities and their genetic regulation in plants subjected to drought and salinity stresses. Int. J. Mol. Sci..

[29] Sun Y, Li P, Deng M, Shen D, Dai G, Yao N, Lu Y (2017). The *Ralstonia solanacearum* effector RipAK suppresses plant hypersensitive response by inhibiting the activity of host catalases. Cell Microbiol..

[30] Raza A, Su W, Gao A, Mehmood SS, Hussain MA, Nie W, Lv Y, Zou X, Zhang X (2021). Catalase (CAT) gene family in rapeseed (*Brassica*
*napus* L.): Genome-wide analysis, identification, and expression pattern in response to multiple hormones and abiotic stress conditions. Int. J. Mol. Sci..

[31] Liu F, Huang N, Wang L, Ling H, Sun T, Ahmad W, Muhammad K, Guo J, Xu L, Gao S, Que Y, Su Y (2018). A novel L-ascorbate peroxidase 6 gene, ScAPX6, plays an important role in the regulation of response to biotic and abiotic stresses in sugarcane. Front. Plant Sci..

[32] Liu J, Wang J, Lee S, Wen R (2018). Copper-caused oxidative stress triggers the 31 activation of antioxidant enzymes via ZmMPK3 in maize leaves. PLoS ONE.

[33] Maiti S, Paul S, Pal A (2012). Isolation, characterization, and structure analysis of a non-TIR-NBS-LRR encoding candidate gene from MYMIV-resistant *Vigna mungo*. Mol. Biotechnol..

[34] Jalmi SK, Bhagat PK, Verma D, Noryang S, Tayyeba S, Singh K, Sharma D, Sinha AK (2018). Traversing the links between heavy metal stress and plant signaling. Front. Plant Sci..

[35] Sheldon AR, Menzies NW (2005). The effect of copper toxicity on the growth and root morphology of Rhodes grass (*Chloris gayana* Knuth.) in resin buffered solution culture. Plant Soil.

[36] Pietrini F, Carnevale M, Beni C, Zacchini M, Gallucci F, Santangelo E (2019). Effect of different copper levels on growth and morpho-physiological parameters in giant reed (*Arundo*
*donax* L.) in semi-hydroponic mesocosm experiment. Water.

[37] Franco A, Buoso S, Zanin L, Pinton R, Tomasi N (2022). Copper toxicity in maize: The severity of the stress is reduced depending on the applied Fe-chelating agent. J. Plant Growth Regul..

[38] Li C, Li Y, Ding C (2019). The role of copper homeostasis at the host-pathogen axis: From bacteria to fungi. Int. J. Mol. Sci..

[39] Adrees M, Ali S, Rizwan M, Ibrahim M, Abbas F, Farid M, Zia-ur-Rehman M, Irshad MK, Bharwana SA (2015). The effect of excess copper on growth and physiology of important food crops: A review. Environ. Sci. Pollut. Res..

[40] Younis ME, Tourky SMN, Elsharkawy SEA (2018). Symptomatic parameters of 27 oxidative stress and antioxidant defense system in *Phaseolus*
*vulgaris* L. in response to copper or cadmium stress. S. Afr. J. Bot..

[41] Audet P, Charest C (2007). Heavy metal phytoremediation from a meta-analytical perspective. Environ. Pollut..

[42] Wairich A, De-Conti L, Lamb TI, Keil R, Neves LO, Brunetto G, Sperotto RA, Ricachenevsky FK (2022). Throwing copper around: How plants control uptake, distribution, and accumulation of copper. Agronomy.

[43] Caverzan A, Casassola A, Brammer SP (2016). Antioxidant responses of wheat plants under stress. Genet. Mol. Biol..

[44] Scandalios JG (2005). Oxidative stress: molecular perception and transduction of signals triggering antioxidant gene defenses. Braz. J. Med. Biol. Res..

[45] Werner T, Motyka V, Laucou V, Smets R, Van Onckelen H, Schmülling T (2003). Cytokinin-deficient transgenic Arabidopsis plants show multiple developmental alterations indicating opposite functions of cytokinins in the regulation of shoot and root meristem activity. Plant Cell.

[46] Kieffer P, Planchon S, Oufir M, Ziebel J, Dommes J, Hoffmann L (2009). Combining proteomics and metabolite analyses to unravel cadmium stress-response in poplar leaves. J. Proteome Res..

[47] Hossain Z, Komatsu S (2013). Contribution of proteomic studies towards understanding plant heavy metal stress response. Front. Plant Sci..

[48] Abawi GS, Corrales P (1990). Seed transmission and effects of fungicide seed treatments against Macrophomina phaseolina in dry edible beans. Turrialba Volumen.

[49] Singh V, Khrab P (2013). SciVerse ScienceDirect A paradigm shift from teaching to learning gross anatomy: Meta-analysis of implications for instructional methods. J. Anat. Soc. India.

[50] Lichtenthaler HK (1987). Chlorophylls and carotenoids: Pigments of photosynthetic biomembranes. Meth. Enzymol..

[51] Nelson N (1944). A photometric adaptation of the Somogyis method for the determination of reducing sugar. Anal. Chem..

[52] Singleton VL, Orthofer R, Lamuela-Raventos RM (1999). Analysis of total phenols and other oxidation substrates and antioxidants by means of Folin-Ciocalteau reagent. Meth. Enzymol..

[53] Lowry OH, Rosebrough NJ, Farr AL, Randall RJ (1951). Protein measurement with the Folin phenol reagent. J. Biol. Chem..

[54] Kumar KB, Khan PA (1982). Peroxidase and polyphenol oxidase in excised ragi (*Eleusine corocana* cv PR 202) leaves during senescence. Indian J. Exp. Biol..

[55] Mayer AM, Harel E, Shaul RB (1965). Assay of catechol oxidase, a critical comparison of methods. Phytochemistry.

[56] Wan CY, Wilkins TA (1994). A modified hot borate method significantly enhances the yield of high-quality RNA from cotton (*Gossypium*
*hirsutum* L.). Anal. Biochem..

[57] International Soil Reference and Information Centre (2002). https://www.isric.org/.

[58] Bose S, Bhattacharyya AK (2008). Heavy metal accumulation in wheat plant grown in soil amended with industrial sludge. Chemosphere.

[59] Chen L, Wan H, Qian J, Guo J, Sun C, Wen J, Yi B, Ma C, Tu J, Song L, Fu T, Shen J (2018). Genome-wide association study of cadmium accumulation at the seedling stage in rapeseed (*Brassica*
*napus* L.). Front. Plant Sci..

