# Supplementary material for: Farmyard manure, a potential organic additive to reclaim copper and Macrophomina phaseolina stress responses in mash bean plants
Source: Sci Rep. 2023 Sep 1;13:14383. doi: 10.1038/s41598-023-41509-3 (PMC10474152; doi:10.1038/s41598-023-41509-3)
Supplement: Supplementary file 1 — Supplementary Figures. [file 41598_2023_41509_MOESM1_ESM.docx]

Supplementary Figure [S1]: Sodium dodecyl sulphate-polyacrylamide gel electrophoresis (SDS-PAGE) for 45-days old mash bean leaf due to the effect of soil amendment with 2% FYM on charcoal rot disease caused by *Macrophomina phaseolina* (MP) and excess copper (Cu) at 45^th^ days of sowing. T_1_: -ve Control; T_2_: + ve Control (MP); T_3_: Cu (50 mg/kg); T_4_: Cu (100 mg/kg); T_5_: Cu (50 mg/kg) + MP; T_6_: Cu (100 mg/kg) + MP; T_7_: 2% FYM; T_8_: 2% FYM + MP; T_9_: 2% FYM + Cu (50 mg/kg); T_10_: 2% FYM + Cu (100 mg/kg); T_11_: 2% FYM + Cu (50 mg/kg)+ MP and T_12_: 2% FYM + Cu (100 mg/kg) + MP. Yellow boxes indicate the grouping of gels cropped from different parts of the same gel.


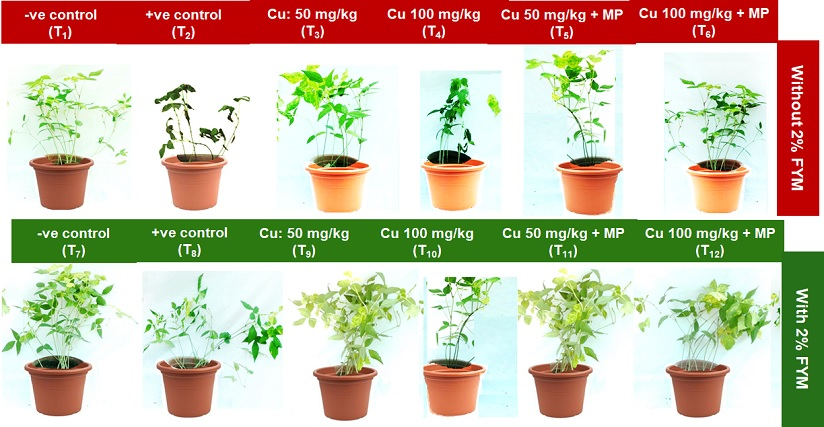


Supplementary Figure [S2]: Effect of soil amendment with 2% FYM on charcoal rot disease caused by *Macrophomina phaseolina* (MP) and excess copper (Cu).
